# Longitudinal Associations Between Anxiety and Depression in Transition From Childhood to Adolescence

**DOI:** 10.1155/da/5659455

**Published:** 2026-07-07

**Authors:** Gizem Keskin, André Plamondon, Suzanne Tough, Sheri Madigan

**Affiliations:** ^1^ Department of Psychology, University of Calgary, Calgary, Alberta, Canada, ucalgary.ca; ^2^ Alberta Children’s Hospital Research Institute, Calgary, Alberta, Canada, albertahealthservices.ca; ^3^ Hotchkiss Brain Institute, Calgary, Alberta, Canada; ^4^ Department of Foundations and Practices in Education, Laval University, Laval, Quebec, Canada, ulaval.ca; ^5^ Cumming School of Medicine, Department of Pediatrics, University of Calgary, Calgary, Alberta, Canada, ucalgary.ca

**Keywords:** anxiety, child development, depression, internalizing problems

## Abstract

**Background:**

The transition from childhood to adolescence is often accompanied by an increase in internalizing problems, particularly anxiety and depression. Although anxiety and depression are conceptually distinct, they also frequently co‐occur. Some evidence suggests that anxiety may precede depression; however, the longitudinal dynamics between the two remain unclear. In the current study, we used a random intercept cross‐lagged panel model (RI‐CLPM) to disentangle between‐person stability from within‐person fluctuations. This approach allows us to examine how changes in anxiety and depression predict each other over time during the transition from childhood to adolescence.

**Methods:**

Participating children (*n* = 1499; 47.8% girls) from the All Our Families (AOF) cohort reported on their anxiety and depression using the Behavior Assessment System for Children – Third Edition (BASC‐3) four times between 2020 and 2023 (mean age at Time 1 = 9.66 years; *SD* = 0.81).

**Results:**

At the between‐person level, which captures stability in the levels of anxiety and depression, there was a strong positive correlation. At the within‐person level, which captures within‐person variations over time, concurrent associations were significant at most time points. However, cross‐lagged effects were unidirectional, with depression predicting anxiety, but not vice versa. These effects were significant from Time 2 to Time 3 and from Time 3 to Time 4 and were consistent across sexes.

**Conclusions:**

The findings underscore the significance of the timing of child‐specific effects between anxiety and depression, indicating that depression, rather than anxiety, may serve as the primary driving factor in the longitudinal association between the two symptom clusters.

## 1. Introduction

It is well‐established that anxiety and depression frequently co‐occur [1, 2], and emerging evidence suggests that anxiety and depression may relate to one another over time [3]. For instance, heightened anxiety may lead to feelings of hopelessness and low self‐worth, core symptoms of depression [4, 5]. Conversely, depression may increase worry and fear, which are central to anxiety [6]. It is also well known that early manifestations of either anxiety or depression are risk factors for adverse mental and physical health outcomes across the lifespan [7, 8].

The transition to adolescence – a period marked by significant biological, social, and psychological changes – heightens vulnerability to mental health difficulties, including increased rates of anxiety and depression [9]. These challenges have been further exacerbated by the COVID‐19 pandemic, which substantively disrupted the daily life and well‐being of children and adolescence [10, 11]. The present study focused on examining the interplay between anxiety and depression symptoms during a developmental period that is particularly sensitive to mental health problems and one that was further intensified by the impacts of the COVID‐19 pandemic. Although this area remains understudied, understanding how anxiety and depression interact over time is important for informing targets of early intervention.

Several theoretical explanations have been proposed to account for the high comorbidity between anxiety and depression [2, 12, 13]. One notable framework is the Tripartite model of anxiety and depression [14], which suggests that anxiety and depression share the common component of negative affect in particular, while also retaining distinct syndrome features such as low positive affect for depression and physiological hyperarousal for anxiety. This model helps to explain both the co‐occurrence and differentiation of symptoms and aligns with evidence that shared features, such as rumination, irritability, restlessness, and attentional problems, contribute to diagnostic overlap [15–17]. Moreover, shared etiological factors, such as genetic predisposition and early experiences (e.g., trauma), may contribute to the co‐development of anxiety and depression [18].

Most previous studies examining the longitudinal associations between childhood anxiety and depression have focused on between‐person differences—that is, whether individuals with higher levels of anxiety tend to show higher levels of depression (and vice versa) compared to others in the sample [19–22]). However, these studies often overlook within‐person dynamics, such as whether an individual experiencing higher anxiety than their own typical level at one time point is more likely to experience elevated depression relative to their own average at a subsequent time point. Since between‐person and within‐person level analyses address different research questions—such as comparing individuals to one another versus examining fluctuations within individuals relative to their own overall level—they may also lead to different conclusions. Hence, it is important to distinguish between‐ and within‐person levels as well as account for between‐person differences when examining within‐person dynamics to better understand the interplay between anxiety and depression across late childhood and early adolescence.

### 1.2. Current Study

In the present study, we accounted for between‐person‐level differences while also examining within‐person dynamics in anxiety and depression using data collected during the pandemic from a sample of 1499 children across four time points between ages 9 and 13. Girls are more likely than boys to report higher anxiety and depressive symptoms [20]. The onset for depression also occurs earlier for girls than boys [23, 24], whereas anxiety onset does not appear to differ by sex [25]. In addition, females are more likely to experience co‐occurring depression and anxiety [26]. Prior research suggests stronger continuity and symptom progression between anxiety and depression among females [27, 28]. Given these patterns, we examined whether associations between anxiety and depression differ by sex over time. Based on existing literature, we hypothesized that

1. Anxiety and depression would show positive bidirectional effects after accounting for between‐person differences.
2. Bidirectional effects between anxiety and depression would be stronger among girls compared to boys.

## 2. Methods

### 2.1. Participants and Procedure

Participants were recruited to participate in the All Our Families (AOF) study, an ongoing pregnancy cohort of mothers and children from Calgary, Canada. Women were recruited between August 2008 and December 2010 through primary healthcare offices, community advertising, and the regional laboratory. Inclusion criteria were (1) > 18 years, (2) fluent in English, (3) gestational age < 24 weeks, and (4) receiving community‐based prenatal care [29, 30]. Mothers completed surveys from pregnancy to child age 8, and both mothers and children completed surveys from ages 9 to 13. The current sample includes 1499 children (% girls = 47.8) who reported their anxiety and depression at least once across four time points: Time 1 (July–August, 2020), Time 2 (March–April, 2021), Time 3 (November 2021–January, 2022), and Time 4 (January–July, 2023). The first three time points occurred during the COVID‐19 pandemic, whereas the fourth occurred during the post‐pandemic period. Public health restrictions at the study site were lifted in June 2022, and the World Health Organization declared the pandemic no longer a public health emergency on May 5, 2023.

Table 1 shows the demographic characteristics of the sample. At Time 1 of the current study, children’s age ranged between 8 and 11 years (*M* = 9.66; *SD* = 0.81), and maternal age ranged between 29 and 54 years (*M* = 41.56; *SD* = 4.35). Table 2 shows anxiety and depression scores across waves and bivariate correlations between them, indicating that both remained relatively stable over time.

**Table 1:** Descriptive statistics of the demographic characteristics.

Variables	*n*	*%*	*M* (*SD*)	Min–max
Child sex				
Girl child	717	47.8	—	—
Boy child	782	52.2	—	—
Maternal ethnicity				
White	1,1191	80.5	—	—
Chinese	80	5.4	—	—
South Asian	44	3.0	—	—
Latin American	29	2.0	—	—
Filipino	21	1.4	—	—
Southeast Asian	16	1.1	—	—
Arab	14	0.9	—	—
Japanese	7	0.5	—	—
Black	13	0.9	—	—
Metis	6	0.4	—	—
First Nations (registered)	2	0.2	—	—
Korean	1	0.1	—	—
West Asian	1	0.1	—	—
Mixed/other	45	3.0	—	—
Missing	8	0.5	—	—
Family income (T1)				
$29,999 or less	25	1.7	—	—
$30,000–$39,999	21	1.4	—	—
$40,000–$49,999	52	3.5	—	—
$50,000–$79,999	71	4.7	—	—
$80,000–$99,999	112	7.5	—	—
$100,000–$124,999	218	14.5	—	—
$125,000–$174,999	256	17.1	—	—
$175,000 or more	363	24.2	—	—
Missing	381	25.4	—	—
Maternal age (T1)	1279	—	41.56 (4.35)	29–54
Child age (T1)	891	—	9.66 (0.81)	8–11
Child age (T2)	1025	—	10.90 (0.78)	9.70–12.60
Child age (T3)	1015	—	11.61 (0.76)	10.40–13.30
Child age (T4)	1333	—	12.82 (0.79)	11.56–14.68

*Note*: Maternal and child age values are in years.

**Table 2:** Descriptive statistics of and bivariate correlations between anxiety and depression.

	*N*	*M* (*SD*)	1	2	3	4	5	6	7	8
1. Anxiety (T1)	884	49.39 (10.84)	—	—	—	—	—	—	—	—
2. Anxiety (T2)	1019	49.00 (11.53)	0.685 ^∗^	—	—	—	—	—	—	—
3. Anxiety (T3)	1022	50.22 (12.65)	0.549 ^∗^	0.698 ^∗^	—	—	—	—	—	—
4. Anxiety (T4)	1294	53.66 (14.28)	0.476 ^∗^	0.564 ^∗^	0.663 ^∗^	—	—	—	—	—
5. Depression (T1)	884	49.42 (9.62)	0.696 ^∗^	0.434 ^∗^	0.375 ^∗^	0.313 ^∗^	—	—	—	—
6. Depression (T2)	1020	49.54 (11.01)	0.520 ^∗^	0.707 ^∗^	0.544 ^∗^	0.429 ^∗^	0.547 ^∗^	—	—	—
7. Depression (T3)	1022	51.34 (13.06)	0.385 ^∗^	0.459 ^∗^	0.725 ^∗^	0.522 ^∗^	0.417 ^∗^	0.604 ^∗^	—	—
8. Depression (T4)	1293	53.88 (15.17)	0.375 ^∗^	0.393 ^∗^	0.514 ^∗^	0.750 ^∗^	0.353 ^∗^	0.454 ^∗^	0.604 ^∗^	—

*Note*: Age‐normed *T*‐scores are reported.

^∗^ *p* < 0.001.

### 2.2. Measures

#### 2.2.1. Anxiety and Depression

Child‐reported anxiety and depression subscales of the Behavior Assessment System for Children – Third Edition (BASC‐3: [31]) were utilized to measure child anxiety (10 items) and depression (10 items) at all time points. We used self‐reports of child anxiety and depression, as children tend to report a greater frequency of internalizing symptoms than parents during the transition from childhood to adolescence, reflecting the nature of internalizing challenges [32]. Items are rated on a 4‐point scale ranging from “Never” to “Almost always.” BASC‐3 has two self‐report versions based on the age range: the child form targets youth aged between 6 and 11 years, and the adolescent form targets youth aged between 12 and 21 years [31], and both self‐report measures have been shown to be reliable and valid among population and clinical samples [33, 34]. We used both versions, as the mean age at Time 1 of the study was 9.66 years (*SD* = 0.81). To ensure comparability of anxiety and depression scores across time and age in the same model, we used age‐standardized *T* scores. Anxiety and depression scores were calculated for each time point and age group (i.e., < 11 years vs ≥12 years) by summing the corresponding items and converting these summed scores to standardized *T*‐scores (i.e., *M* = 50, *SD* = 10) based on age norms. A *T*‐score of ≥70 suggests a clinically significant level of anxiety and/or depression. The anxiety and depression subscales also showed high internal consistency across time points and age groups (*αs* = 0.89–0.94 for anxiety and 0.83–0.93 for depression, respectively).

#### 2.2.2. Child Sex

Child biological sex was reported by caregivers as a binary measure (0 = *male*; 1 = *female*) 4 months postpartum.

### 2.3. Analytical Strategy

**Figure 1:**
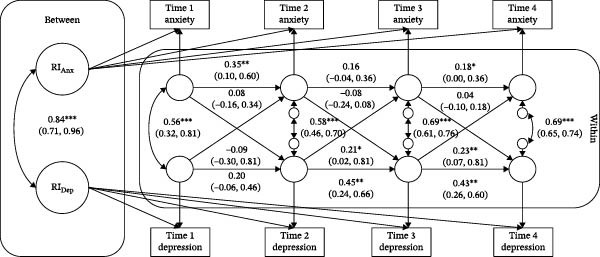
RI‐CLPM of anxiety and depression (Model 1). *Note*. RI_Anx_ = Random intercept of anxiety; RI_Dep_ = Random intercept of depression; *p* < 0.05 ^∗^; *p* < 0.01 ^∗∗^; *p* < 0.001 ^∗∗∗^; Standardized estimates are reported. Confidence intervals are reported in brackets.

Descriptive analyses were performed in R (RStudio [35]; Text S1 and Tables 1 and 2) using the *lavaan* package [36]. Using the MLR estimator in our structural equation models, missing data was dealt with full information maximum likelihood [37]. To reach our research objectives, we ran RI‐CLPM [38] in Mplus [39], which disentangles between‐ and within‐person variance and allows for the examination of within‐person level associations after accounting for between‐person differences [38]. In Model 1 (Figure 1), we estimated random intercepts for anxiety and depression by regressing each onto its respective random intercepts, with the regression paths set to 1. The correlation between variances of the random intercepts of anxiety and depression were then estimated. We centered anxiety and depression at the within‐person level, and estimated autoregressive and cross‐lagged paths between them. We also included concurrent correlations for variances or residual variances of within‐person centered anxiety and depression. Further, we correlated the variance of random intercepts of anxiety and depression with Time 1 variances of the within‐person anxiety and depression, respectively to overcome the stationary assumption of the RI‐CLPM (Text S2 for a detailed discussion of the stationary assumption in RI‐CLPMs, see Anderson [40]).

#### 2.3.1. Role of Sex Differences

To assess the moderating effect of child sex, we conducted a multigroup RI‐CLPM in Model 2 (Text S3). To test Hypothesis 2, we first constrained across sex groups cross‐lagged effects from depression to anxiety in Model 3 (Text S4), and from anxiety to depression in Model 4 (Text S4). Then, we conducted chi‐square difference tests with Satorra–Bentler correction [41, 42] to compare model fit between Model 2 and Model 3 and between Model 2 and Model 4.

### 2.4. Transparency and Openness

Analysis code is provided in the supplemental materials (Texts S1–S4). Hypotheses and analyses were pre‐registered on the open science framework (OSF) prior to data access and analysis (https://osf.io/ku3tq). Our initial analytical strategy was to employ the Bivariate Latent Change Score Model (BLCSM); however, we encountered convergence issues, which is likely due to too few time points for this approach [43]. Thus, we implemented RI‐CLPM instead [38]. Our hypotheses remained unchanged. Further, instead of the mean values, we used the *T*‐scores for the anxiety and depression subscales as suggested by the developers of the Behavior Assessment System for Children ‐ Third Edition [44].

## 3. Results

### 3.1. Descriptive Statistics

Table 2 illustrates that mean *T* scores of anxiety and depression were relatively stable across time. Further, bivariate correlations showed that anxiety and depression were positively and significantly correlated concurrently and longitudinally with each other (Table 2).

### 3.2. Longitudinal Associations Between Anxiety and Depression

In Model 1 (Figure 1), fit indices indicated a good model fit for the RI‐CLPM (Table S1 Model 1). The correlation between random intercepts of anxiety and depression was positive and very large at the between‐person level (*r* = 0.84). Therefore, children who overall showed a greater level of anxiety than other children were also likely to show a greater level of depression than other children across time. At the within‐person level, concurrent associations between (residual) variances of anxiety and depression were positive and significant at each time point (*r* = 0.56–0.69).

Autoregressive effects were mostly positive. Specifically, for anxiety, the effect size of the autoregressive was twice as large between initial time points (i.e., from Time 1 to Time 2: *β* = 0.35) than later time points (i.e., *β* = 0.16–0.18), whereas for depression, it was half as small between initial points (i.e., from T1 to T2 [*β* = 0.20]) compared to later time points (i.e., *β* = 0.43–0.45).

While we hypothesized that bidirectional cross‐lagged paths would be present (Hypothesis 1), this was not supported. Rather, unidirectional cross‐lagged paths were uncovered from depression to anxiety from both T2 to T3 (*β* = 0.21) and T3 to T4 (*β* = 0.23; Figure 1). Thus, when children experienced a greater level of depression than they usually experienced, they were also more likely to experience a greater level of anxiety at a later time point.

### 3.3. Moderating Role of Sex

In Model 2, we conducted a multigroup RI‐CLPM with child sex as the grouping variable but allowed all parameters to be freely estimated. Model fit indices indicated a good model fit (Table S1 Model 2). In Model 3, we constrained cross‐lagged effects from depression to anxiety, which did not result in worse model fit (Table S1). In Model 4, we constrained cross‐lagged effects from anxiety to depression, which did not result in worse model fit (Table S1). Results rejected Hypothesis 2 because the cross‐lagged paths from anxiety to depression and from depression to anxiety did not differ across sex groups. Hence, we reported the results from Model 1 in Figure 1 as the final model.

## 4. Discussion

We investigated the potential bidirectional effects between anxiety and depression in the transition from childhood to adolescence, with data collection during and beyond the pandemic, and whether these bidirectional effects are stronger for girls than for boys. Using a RI‐CLPM approach, we accounted for between‐person differences when examining within‐person effects. Our findings are partially consistent with the Tripartite model of anxiety and depression [14], as there was a strong positive correlation at the between‐person level between anxiety and depression. However, our findings showed that the longitudinal associations between anxiety and depression were unidirectional. Specifically, fluctuations in depression in a year positively predicted fluctuations in anxiety next year, while the inverse effects of anxiety on depression were not significant. Further, girls and boys did not differ in terms of the magnitude of the prospective effects of anxiety and depression on each other.

The prospective and positive effect of depression on anxiety may reflect the close link between depressive symptoms and social withdrawal in childhood and adolescence. Withdrawal during social interactions can elicit negative peer feedback, which in turn can increase anxiety [45]. However, the absence of cross‐lagged effects from anxiety to depression contrasts with prior research [19, 46]. One potential explanation is that depression amplifies emotional vulnerabilities, such as fear and worry, that are more characteristic of anxiety disorders [6]. The observed directionality may also reflect the broader pandemic context, during which children experienced elevated levels of health‐related and social anxiety [47, 48]. These effects may have been particularly pronounced among children with pre‐existing mental health conditions [49], who may have been more vulnerable to pandemic‐related stress.

Additionally, the autoregressive paths were stronger for depression than for anxiety, leaving less residual variance in depression to be explained by anxiety. This finding aligns with developmental patterns in the current sample’s age period, as the age of onset is typically earlier for anxiety than for depression [13]. Taken together, the non‐significant pathway from anxiety to depression does not necessarily imply the absence of an effect; rather, it may reflect the relatively stronger predictive role of depression within this specific developmental and contextual context.

Contrary to our expectations, we did not observe sex differences in cross‐lagged effects, suggesting that girls and boys showed similar patterns in the prospective associations between anxiety and depression in the transition from late childhood to early adolescence. Previous research using between‐person models suggests that sex differences in anxiety and depression tend to become more pronounced later in adolescence, with girls showing increases in internalizing problems over time [28]. Therefore, late childhood to early adolescence may not be a sensitive developmental window to detect sex‐specific differences in the longitudinal associations between anxiety and depression. Another possible explanation is that girls and boys may exhibit similar patterns due to prominent internalizing problems observed during the COVID‐19 pandemic [50].

The long‐term, or crystallized characteristics of anxiety and depression were positively and robustly correlated at the between‐person level. This suggests that children who tended to report higher levels of anxiety also tended to report higher levels of depression across the study period. Moreover, at the within‐person level, concurrent associations showed that when a child experienced higher anxiety than their own typical level, they were also likely to report elevated depression at the same time (for three out of four time points). These consistent positive correlations at both the between‐ and within‐person levels align with theory [14] and decades of research showing a strong association between anxiety and depression in children and adolescence [2, 51]. Future research could use longitudinal models to disaggregate shared symptoms of anxiety and depression, such as negative affect [14]—at both the between‐ and within‐person levels, to clarify which dimensions of distress contribute most to the co‐occurrence between anxiety and depression.

## 5. Limitations

Certain limitations of our study are noteworthy. First, our study relied on a community sample. Prior research suggests that internalizing symptom levels tend to be elevated in clinical samples compared to community samples [52–54]. That said, pandemic‐related meta‐analytic research on mental health before and during the pandemic suggests that, regardless of baseline symptom levels (i.e., clinical, threshold, subthreshold, and low), most children experienced increases in depression [10], while literature showed mixed evidence regarding anxiety [55]. Nonetheless, the observed associations in this study should be explored in clinical populations.

Second, the majority of participants identified as White, all participants were proficient in English, and participants were mostly from families with high household income. Hence, our findings may not capture the experiences of children from different sociocultural and socioeconomic backgrounds. Considering that (1) individuals with lower socioeconomic status may have limited access to mental health resources; and (2) marginalized identities, with respect to ethnicity, language, and cultural differences, may be at greater risk for mental health difficulties due to discriminatory policies and practices [56–58], future research should prioritize socioeconomically, ethnically, and culturally diverse samples to better understand the dynamics between anxiety and depression during the transition from childhood to adolescence.

Lastly, data collection coincided with fluctuating COVID‐19 restrictions, which may have introduced variability in symptom patterns due to pandemic‐related stress. However, mean levels of anxiety and depression increased across time points, which is in line with the previous findings that internalizing problems are likely to increase from early to mid‐adolescence [46]. Further, the age range of the adolescents in the current study may result in missing the onset of internalizing difficulties for adolescents who develop anxious and depressive symptoms later in adolescence. Thus, future studies may benefit including a greater number of time points to examine the co‐development of anxiety and depression across adolescence. Relatedly, the 1‐year time interval may not adequately capture fluctuations in anxiety and depression. Therefore, additional longitudinal studies are needed to clarify the developmental dynamics between anxiety and depression across childhood and adolescence, particularly under varying contextual conditions and with shorter intervals between observations.

## 6. Conclusions

Anxiety and depression frequently co‐occur in childhood and adolescence. Findings suggest that in emerging adolescence, depression may precede the onset of anxiety. Therefore, strategies that identify the emergence of depression with appropriate follow‐up, including strategies to address emotional concerns, may reduce or mitigate the risk for subsequent mental health concerns. Previous studies have highlighted that anxiety and depression are likely to co‐occur, and our findings suggested a temporal ordering of depression to anxiety. These findings underscore the importance of early interventions aimed at promoting emotional well‐being in middle childhood and adolescence.

## Funding

This study was funded by the Alberta Innovates Interdisciplinary Team (Grant 200700595), the Children and Screens: Institute of Digital Media and Child Development Inc. (Grant CSCOVID002/1055552‐1), and the Social Sciences and Humanities Research Council of Canada Postdoctoral Fellowship (Grant 756‐2025‐0653).

## Conflicts of Interest

The authors declare no conflicts of interest.

## Supporting Information

Additional supporting information can be found online in the Supporting Information section.

## Supporting information


**Supporting Information** Supplemental materials include the following: 1. Table S1: Model Fit Indices and Model Comparisons for RI‐CLPMs of Anxiety and Depression 2. Figure S1: RI‐CLPM of Anxiety and Depression: Results for Girls (Model 2) 3. l Figure S2: RI‐CLPM of Anxiety and Depression: Results for Boys (Model 2) 4. Text S1: R Script for Descriptive Statistics 5. Text S2: Mplus Syntax for RI‐CLPM of Anxiety and Depression 6. Text S3: Mplus Syntax for Multigroup RI‐CLPM of Anxiety and depression based on Child Sex 7. Text S4: Mplus Syntax for Constrained Cross‐Lagged Paths Between Depression and Anxiety in Model 3 and Model 4.

## Data Availability

Data sharing is not applicable to this article, as the data is subject to third‐party restrictions due to sensitive information.
