# Supplementary material for: Longitudinal Associations Between Anxiety and Depression in Transition From Childhood to Adolescence
Source: Depress Anxiety. 2026 Jul 7;2026:5659455. doi: 10.1155/da/5659455 (PMC13338837; doi:10.1155/da/5659455)
Supplement: Supplementary file 1 — Supporting Information Supplemental materials include the following: 1. Table S1: Model Fit Indices and Model Comparisons for RI‐CLPMs of Anxiety and Depression 2. Figure S1: RI‐CLPM of Anxiety and Depression: Results for Girls (Model 2) 3. l Figure S2: RI‐CLPM of Anxiety and Depression: Results for Boys (Model 2) 4. Text S1: R Script for Descriptive Statistics 5. Text S2: Mplus Syntax for RI‐CLPM of Anxiety and Depression 6. Text S3: Mplus Syntax for Multigroup RI‐CLPM of Anxiety and depression based on Child Sex 7. Text S4: Mplus Syntax for Constrained Cross‐Lagged Paths Between Depression and Anxiety in Model 3 and Model 4. [file DA-2026-5659455-s001.docx]

**Supplemental Table 1**

*Model Fit Indices and Model Comparisons for RI-CLPMs of Anxiety and Depression*

|  |  |  |  |  |  | *RMSEA* | |  |  |  |  |  |  |  |
| --- | --- | --- | --- | --- | --- | --- | --- | --- | --- | --- | --- | --- | --- | --- |
|  | *df* | χ² | *Scf* | *CFI* | *TLI* | *estimate* | *95% CI* | Comparison | Δ *df* | *TRd* | *p* | Δ*CFI* | Δ*TLI* | Δ*RMSEA* |
| Model 1 | 5 | 1.59 | 1.28 | .997 | .983 | .000 | [.017, .059] |  |  |  |  |  |  |  |
| Model 2 | 10 | 8.34 | 1.21 | 1.000 | 1.000 | .000 | [.000, .035] |  |  |  |  |  |  |  |
| Model 3 | 13 | 9.95 | 1.26 | 1.000 | 1.000 | .000 | [.000, .028] | Model 2-3 | 1.71 | 3 | .634 | 0 | 0 | 0 |
| Model 4 | 13 | 9.29 | 1.24 | 1.000 | 1.000 | .000 | [.000, .026] | Model 2-4 | 1.07 | 3 | .785 | 0 | 0 | 0 |

*Notes*. Scf = Scaling Correction Factor for MLR. Chi-squared test of model fit values are reported. TRd = Sattora-Bentler scaled Chi-square difference.

**Supplemental Figure 1**

*RI-CLPM of Anxiety and Depression: Results for Girls (Model 2)*

Time 4 Anxiety

Time 4 Depression

Time 3 Depression

Time 3 Anxiety

Time 2 Depression

Time 2 Anxiety

Time 1 Anxiety

**Between**

.14

[-.18, .45]

.21

[-.13, .54]

.32

[-.10, .73]

.05

[-.28, .91]

-.03

[-.41, .36]

.64**

[.18, 1.09]

.79***

[.58, 1.01]

.36

[-.24, .95]

.40***

[.18, .62]

**Within**

1.01***

[.78, 1.23]

-.03

[-.39, .32]

.29*

[.06, .52]

.28*

[.06, .50]

.35

[-.04, .73]

.51*

[.22, .81]

.60**

[.28, .91]

Time 1 Depression

*Notes.* RI_Anx_ = Random Intercept of Anxiety; RI_Dep_ = Random Intercept of Depression; *p* < .05*; *p* < .01**; *p* < .001***; Unstandardized estimates are reported.

**Supplemental Figure 2**

*RI-CLPM of Anxiety and Depression: Results for Boys (Model 2)*

Time 4 Anxiety

Time 4 Depression

Time 3 Depression

Time 3 Anxiety

Time 2 Depression

Time 2 Anxiety

Time 1 Anxiety

**Between**

.25

[-.11, .61]

.26

[-.10, .62]

.43**

[.17, .68]

-.05

[-.33, .23]

-.19

[-.48, .11]

.24

[-.10, .58]

.37**

[.19, .56]

.30**

[.09, .50]

.29*

[.02, .56]

.42**

[.12, .73]

**Within**

.18

[-.02, .37]

.10

[-.19, .39]

-.23

[-.50, .05]

.58***

[.43, .73]

.52***

[.29, .78]

-.00

[-.45, .44]

.57**

[.24, .89]

Time 1 Depression

*Notes.* RI_Anx_ = Random Intercept of Anxiety; RI_Dep_ = Random Intercept of Depression *p* < .05*; *p* < .01**; *p* < .001***; Unstandardized estimates are reported.

**Supplemental Text 1**

*R Script for Descriptive Statistics*

##prep

#install.packages("lavaan")

library(lavaan)

## This is lavaan 0.6-19

## lavaan is FREE software! Please report any bugs.

df <- read.csv("anx_dep_final.csv")

df[df == -999] <- NA

df <- df[,-c(1,2)]

##correlations

model <- "

ycv1basc_anx_t1 ~~ ycv1basc_dep_t1

ycv1basc_anx_t1 ~~ ycv2basc_anx_t1

ycv1basc_anx_t1 ~~ ycv2basc_dep_t1

ycv1basc_anx_t1 ~~ ycv3basc_anx_t1

ycv1basc_anx_t1 ~~ ycv3basc_dep_t1

ycv1basc_anx_t1 ~~ yq9basc_anx_t1

ycv1basc_anx_t1 ~~ yq9basc_dep_t1

ycv1basc_dep_t1 ~~ ycv2basc_anx_t1

ycv1basc_dep_t1 ~~ ycv2basc_dep_t1

ycv1basc_dep_t1 ~~ ycv3basc_anx_t1

ycv1basc_dep_t1 ~~ ycv3basc_dep_t1

ycv1basc_dep_t1 ~~ yq9basc_anx_t1

ycv1basc_dep_t1 ~~ yq9basc_dep_t1

ycv2basc_anx_t1 ~~ ycv2basc_dep_t1

ycv2basc_anx_t1 ~~ ycv3basc_anx_t1

ycv2basc_anx_t1 ~~ ycv3basc_dep_t1

ycv2basc_anx_t1 ~~ yq9basc_anx_t1

ycv2basc_anx_t1 ~~ yq9basc_dep_t1

ycv2basc_dep_t1 ~~ ycv3basc_anx_t1

ycv2basc_dep_t1 ~~ ycv3basc_dep_t1

ycv2basc_dep_t1 ~~ yq9basc_anx_t1

ycv2basc_dep_t1 ~~ yq9basc_dep_t1

ycv3basc_anx_t1 ~~ ycv3basc_dep_t1

ycv3basc_anx_t1 ~~ yq9basc_anx_t1

ycv3basc_anx_t1 ~~ yq9basc_dep_t1

ycv3basc_dep_t1 ~~ yq9basc_anx_t1

ycv3basc_dep_t1 ~~ yq9basc_dep_t1

yq9basc_anx_t1 ~~ yq9basc_dep_t1"

fit <- sem(model, data = df)

summary(fit, standardized = TRUE)

##r values

cov_matrix <- fitted(fit)$cov

std_devs <- sqrt(diag(fitted(fit)$cov))

corr_matrix <- cov_matrix / (std_devs %*% t(std_devs))

corr_matrix

**Supplemental Text 2**

*Mplus Syntax for RI-CLPM of Anxiety and Depression*

VARIABLE: NAMES ARE

id

sex

anx1

dep1

anx2

dep2

anx3

dep3

anx4

dep4;

USEVARIABLES ARE

anx1 anx2 anx3 anx4

dep1 dep2 dep3 dep4;

MISSING ARE ALL (-999);

ANALYSIS:

ESTIMATOR = MLR;

MODEL:

! Create two individual factors (random intercepts)

RIanx BY anx1@1 anx2@1 anx3@1 anx4@1;

RIdep BY dep1@1 dep2@1 dep3@1 dep4@1;

! Create within-person centered variables

canx1 BY anx1@1; canx2 BY anx2@1; canx3 BY anx3@1; canx4 BY anx4@1;

cdep1 BY dep1@1; cdep2 BY dep2@1; cdep3 BY dep3@1; cdep4 BY dep4@1;

! Constrain the measurement error variances to zero

anx1-dep4@0;

! Estimate the lagged effects between

! the within-person centered variables

canx2 ON canx1 cdep1; canx3 ON canx2 cdep2; canx4 ON canx3 cdep3;

cdep2 ON canx1 cdep1; cdep3 ON canx2 cdep2; cdep4 ON canx3 cdep3;

! Estimate the covariance between the within-person

! centered variables at the first wave

canx1 WITH cdep1;

! Estimate the covariances between the residuals of

! the within-person centered variables (the innovations)

canx2 WITH cdep2; canx3 WITH cdep3; canx4 WITH cdep4;

OUTPUT: STDYX SAMPSTAT;

**Supplemental Text 3**

*Mplus Syntax for Multigroup RI-CLPM of Anxiety and depression based on Child Sex*

VARIABLE: NAMES ARE

id

sex

anx1

dep1

anx2

dep2

anx3

dep3

anx4

dep4;

USEVARIABLES ARE

anx1 anx2 anx3 anx4

dep1 dep2 dep3 dep4

sex;

MISSING ARE ALL (-999);

GROUPING = sex (1=boy 2=girl);

DEFINE: !to overcome convergence issues

anx1 = anx1/10;

anx2 = anx2/10;

anx3 = anx3/10;

anx4 = anx4/10;

dep1 = dep1/10;

dep2 = dep2/10;

dep3 = dep3/10;

dep4 = dep4/10;

ANALYSIS:

MODEL:

! Create two individual factors (random intercepts)

RIanx BY anx1@1 anx2@1 anx3@1 anx4@1;

RIdep BY dep1@1 dep2@1 dep3@1 dep4@1;

! Create within-person centered variables

canx1 BY anx1@1; canx2 BY anx2@1; canx3 BY anx3@1; canx4 BY anx4@1;

cdep1 BY dep1@1; cdep2 BY dep2@1; cdep3 BY dep3@1; cdep4 BY dep4@1;

! Constrain the measurement error variances to zero

anx1-dep4@0;

! Estimate the lagged effects between

! the within-person centered variables

canx2 ON canx1 cdep1; canx3 ON canx2 cdep2; canx4 ON canx3 cdep3;

cdep2 ON canx1 cdep1; cdep3 ON canx2 cdep2; cdep4 ON canx3 cdep3;

! Estimate the covariance between the within-person

! centered variables at the first wave

canx1 WITH cdep1;

! Estimate the covariances between the residuals of

! the within-person centered variables (the innovations)

canx2 WITH cdep2; canx3 WITH cdep3; canx4 WITH cdep4;

! Fix the correlation between the individual factors and the other

!Estimate the correlation between random intercepts

RIanx WITH RIdep;

MODEL GIRL:

! Overrule multiple group factor analysis default of equal intercepts

! across groups

[anx1-dep4];

! Overrule multiple group factor analysis default of free latent means

! in second group

[canx1-cdep4@0];

[RIanx@0]; [RIdep@0];

OUTPUT: STDYX SAMPSTAT;

**Supplemental Text 4**

*Mplus Syntax for Constrained Cross-Lagged Paths Between Depression and Anxiety in Model 3 and Model 4*

!Cross-lagged paths from Depression to Anxiety (Model 3)

VARIABLE: NAMES ARE

id

sex

anx1

dep1

anx2

dep2

anx3

dep3

anx4

dep4;

USEVARIABLES ARE

anx1 anx2 anx3 anx4

dep1 dep2 dep3 dep4

sex;

MISSING ARE ALL (-999);

GROUPING = sex (1=boy 2=girl);

DEFINE:

anx1 = anx1/10;

anx2 = anx2/10;

anx3 = anx3/10;

anx4 = anx4/10;

dep1 = dep1/10;

dep2 = dep2/10;

dep3 = dep3/10;

dep4 = dep4/10;

ANALYSIS:

ESTIMATOR = MLR;

MODEL:

! Create two individual factors (random intercepts)

RIanx BY anx1@1 anx2@1 anx3@1 anx4@1;

RIdep BY dep1@1 dep2@1 dep3@1 dep4@1;

! Create within-person centered variables

canx1 BY anx1@1; canx2 BY anx2@1; canx3 BY anx3@1; canx4 BY anx4@1;

cdep1 BY dep1@1; cdep2 BY dep2@1; cdep3 BY dep3@1; cdep4 BY dep4@1;

! Constrain the measurement error variances to zero

anx1-dep4@0;

! Estimate the lagged effects between

! the within-person centered variables

canx2 ON canx1;

canx2 ON cdep1;

canx3 ON canx2;

canx3 ON cdep2;

canx4 ON canx3;

canx4 ON cdep3;

cdep2 ON canx1;

cdep2 ON cdep1;

cdep3 ON canx2;

cdep3 ON cdep2;

cdep4 ON canx3;

cdep4 ON cdep3;

! Estimate the covariance between the within-person

! centered variables at the first wave

canx1 WITH cdep1;

! Estimate the covariances between the residuals of

! the within-person centered variables (the innovations)

canx2 WITH cdep2; canx3 WITH cdep3; canx4 WITH cdep4;

! Fix the correlation between the individual factors and the other

!Estimate the correlation between random intercepts

RIanx WITH RIdep;

Model boy:

canx2 ON cdep1 (a);

canx3 ON cdep2 (b);

canx4 ON cdep3 (c);

MODEL GIRL:

! Overrule multiple group factor analysis default of equal intercepts

! across groups

[anx1-dep4];

! Overrule multiple group factor analysis default of free latent means

! in second group

[canx1-cdep4@0];

[RIanx@0]; [RIdep@0];

canx2 ON cdep1 (a);

canx3 ON cdep2 (b);

canx4 ON cdep3 (c);

OUTPUT: STDYX SAMPSTAT;

!Cross-lagged paths from Anxiety to Depression (Model 4)

VARIABLE: NAMES ARE

id

sex

anx1

dep1

anx2

dep2

anx3

dep3

anx4

dep4;

USEVARIABLES ARE

anx1 anx2 anx3 anx4

dep1 dep2 dep3 dep4

sex;

MISSING ARE ALL (-999);

GROUPING = sex (1=boy 2=girl);

DEFINE:

anx1 = anx1/10;

anx2 = anx2/10;

anx3 = anx3/10;

anx4 = anx4/10;

dep1 = dep1/10;

dep2 = dep2/10;

dep3 = dep3/10;

dep4 = dep4/10;

ANALYSIS:

ESTIMATOR= MLR;

MODEL:

! Create two individual factors (random intercepts)

RIanx BY anx1@1 anx2@1 anx3@1 anx4@1;

RIdep BY dep1@1 dep2@1 dep3@1 dep4@1;

! Create within-person centered variables

canx1 BY anx1@1; canx2 BY anx2@1; canx3 BY anx3@1; canx4 BY anx4@1;

cdep1 BY dep1@1; cdep2 BY dep2@1; cdep3 BY dep3@1; cdep4 BY dep4@1;

! Constrain the measurement error variances to zero

anx1-dep4@0;

! Estimate the lagged effects between

! the within-person centered variables

canx2 ON canx1;

canx2 ON cdep1;

canx3 ON canx2;

canx3 ON cdep2;

canx4 ON canx3;

canx4 ON cdep3;

cdep2 ON canx1;

cdep2 ON cdep1;

cdep3 ON canx2;

cdep3 ON cdep2;

cdep4 ON canx3;

cdep4 ON cdep3;

! Estimate the covariance between the within-person

! centered variables at the first wave

canx1 WITH cdep1;

! Estimate the covariances between the residuals of

! the within-person centered variables (the innovations)

canx2 WITH cdep2; canx3 WITH cdep3; canx4 WITH cdep4;

! Fix the correlation between the individual factors and the other

!Estimate the correlation between random intercepts

RIanx WITH RIdep;

Model boy:

cdep2 ON canx1(a);

cdep3 ON canx2 (b);

cdep4 ON canx3 (c);

MODEL GIRL:

! Overrule multiple group factor analysis default of equal intercepts

! across groups

[anx1-dep4];

! Overrule multiple group factor analysis default of free latent means

! in second group

[canx1-cdep4@0];

[RIanx@0]; [RIdep@0];

cdep2 ON canx1(a);

cdep3 ON canx2 (b);

cdep4 ON canx3 (c);

OUTPUT: STDYX SAMPSTAT;
